# Osteosarcoma Associated With Diamond-Blackfan Anaemia: A Case of a Child Receiving Growth Hormone Therapy

**DOI:** 10.1080/13577140410001679266

**Published:** 2004-03

**Authors:** Robert S. Lee, Deborah Higgs, Omar Haddo, Jean Pringle, Timothy W. R. Briggs

**Affiliations:** Royal National Orthopaedic Hospital NHS Trust Brockley Hill Stanmore Middx HA7 4LP UK

## Abstract

*Purpose*: Diamond–Blackfan anaemia (DBA) is a rare pure congenital red cell aplasia, usually presenting in infancy or early childhood. The literature suggests a predisposition to haemopoietic malignancy but in addition solid tumours have been reported, with five cases of osteosarcoma described.

*Patient*: A sixth case of a 12-year-old girl with DBA who developed an osteosarcoma of the distal femur is presented.

*Results*: She was treated with methotrexate followed by tumour excision and distal femoral replacement. The patient is currently alive with multiple pulmonary metastases.

*Discussion*: We discuss the association between the administration of growth hormone and future development of malignancy in patients with DBA.


*Sarcoma*, March 2004, *VOL. 8, NO. 1, 47–49*

Taylor & Francis healthsciences

**CASE REPORT**

### Osteosarcoma associated with Diamond-Blackfan anaemia: a case of a child receiving growth hormone therapy

ROBERT S. LEE, DEBORAH HIGGS, OMAR HADDO, JEAN PRINGLE & TIMOTHY W.R. BRIGGS

*Royal National Orthopaedic Hospital NHS Trust, Brockley Hill, Stanmore, Middx HA7 4LP, England*

**Abstract**

*Purpose*: Diamond–Blackfan anaemia (DBA) is a rare pure congenital red cell aplasia, usually presenting in infancy or early childhood. The literature suggests a predisposition to haemopoietic malignancy but in addition solid tumours have been reported, with five cases of osteosarcoma described.

*Patient*: A sixth case of a 12-year-old girl with DBA who developed an osteosarcoma of the distal femur is presented.

*Results*: She was treated with methotrexate followed by tumour excision and distal femoral replacement. The patient is currently alive with multiple pulmonary metastases.

*Discussion*: We discuss the association between the administration of growth hormone and future development of malignancy in patients with DBA.

### Introduction

Diamond–Blackfan anaemia (DBA) is a rare disorder characterised by congenital deficiency of red blood cell precursors resulting in pure red cell aplasia. Although the risk of malignancy in Diamond–Blackfan anaemia is unknown, there are a number of reported cases. A recent review of the literature[1] reveals the majority to be haemopoietic in origin, namely Hodgkin's disease, acute lymphoblastic leukaemia (ALL) and acute myeloid leukaemia (AML). Solid tumours have also been reported, including hepatocellular carcinoma and breast carcinoma. Five cases of osteosarcoma have been documented in the literature and it has been speculated that there is an association between DBA and osteosarcoma.[1–3] We present a sixth case of DBA, who is still alive, and discuss the similarities between our case and those presented previously.

### Case report

A caucasian girl, the second of three children, was born in 1988 after a normal pregnancy, labour and delivery. Concern arose after 3–4 months as she was noted to be lethargic and off her food. By the age of 8 months, her slow growth rate was noted as was her anaemic appearance. Her haemoglobin level was subsequently discovered to be 3.5 g/dl and bone marrow examination revealed erythroid hypoplasia, with all other cell lines being normal. A diagnosis of Diamond–Blackfan anaemia was made.

She presented with many of the typical dysmorphic features associated with the syndrome, namely short stature, short neck, microcephaly, micrognathia, tow-coloured hair, wide set eyes and snub nose. Both her thenar eminences were flattened but her thumbs were of normal appearance and structure. She had no ocular or renal anomalies.

She was initially treated with only low-dose corticosteroids to good effect. It was only from the age of 8 years that she required regular red cell transfusions with a view to attempting to increase her growth velocity.

She subsequently developed iron overload and was treated with subcutaneous desferrioxamine from the age of 11 years. This was not well tolerated due to needle phobia and after approximately 11 months was reverted to steroid treatment, initially at 2 mg/kg reducing to 1 mg/kg. At about the same time, she was commenced on growth hormone to treat her short stature.

Six weeks after the start of this treatment she developed intermittent pain in her right thigh. Initially this

Correspondence to: Robert S. Lee, c/o Timothy Briggs, Royal National Orthopaedic Hospital NHS Trust, Brockley Hill, Stanmore, Middx, England. Fax: +44-20-8909-5709; E-mail: leerobert9@hotmail.com

1357-714X print/1369–1643 © 2004 Taylor & Francis Ltd
DOI: 10.1080/13577140410001679266

was thought to be related to her injections of growth hormone in her right leg and as a result this was discontinued. However, examination revealed a firm selling at the distal end of her right femur. A plain X-ray showed changes consistent with osteosarcoma. Magnetic resonance imaging confirmed the presence of the tumour with no other lesions. A CT chest was clear and and isotope bone scan showed no evidence of metastases.

A needle biopsy, performed in April 2001, confirmed an osteoblastic osteosarcoma. Prior to surgery she received a course of chemotherapy, and in July 2001 (4 months after presentation) she underwent tumour excision (Fig. 1) and had a right distal femoral replacement.

Histology confirmed osteoblastic osteosarcoma with a category 1 (99%) response to pre-operative chemotherapy. The remaining 1% of tumour showed moderate damage. The circumferential margin was focally marginal.

Unforunately she subsequently developed multiple pulmonary metastases requiring thoracoscopic resection in January and May 2002. Despite two courses of intravenous methotrexate, she developed more pulmonary metastases.

In October 2002 she developed sepsis of her distal femoral replacement and underwent open debridement. She is currently alive with multiple pulmonary metastases.

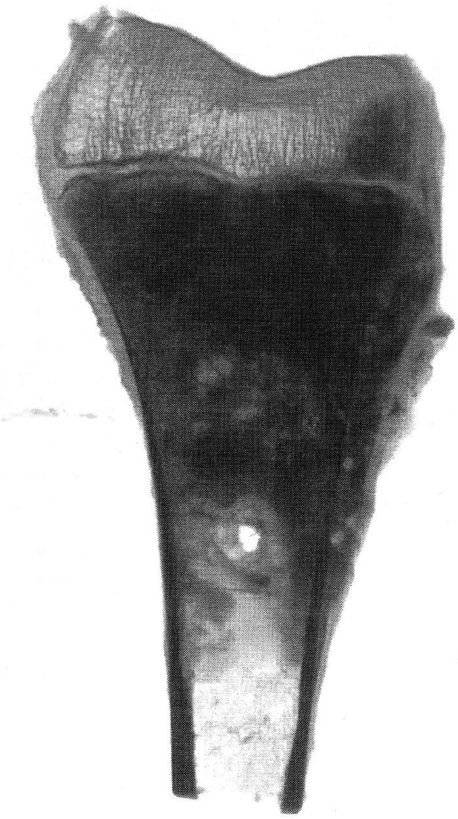

**Fig. 1.** *Digitalised X-ray image of a 4-mm slice through the proximal tibia showing the osteosarcoma lesion.*

### Discussion

Diamond–Blackfan anaemia (DBA) is a rare condition with only approximately 400 cases worldwide. The majority arise from sporadic mutations, though in 10% there is a familial link. Our patient exhibited the typical clinical features of DBA. It is well documented that DBA is associated with myeloid malignancies, with over 14 cases documented in the literature.[1] However, the link between DBA and osteosarcoma is still tenuous with only five previous cases.[1–3] Our case is the sixth such patient with osteosarcoma and is the only one who is still alive and receiving treatment. Of the five previously reported cases, three died of metastatic pulmonary disease and two died of sepsis, with a mean survival of 1.6 years from the time of diagnosis.

The age of presentation of our patient is in line with the mean age of presentation of the other reported cases (12 vs. 11 years; range 5–22 years). Similarly, all those who developed osteosarcoma were transfusion dependent. One worrying feature is that our patient is the third out of the six cases to have developed osteosarcoma following growth hormone treatment. Lipton *et al.*[1] initially questioned whether growth hormone played a role in the development of osteosarcoma in those with DBA. In a recent study of 16 children[4] who received growth hormone treatment for intra-uterine growth retardation, one girl developed an osteosarcoma of her left tibia aged 9 years. A review of the literature also revealed a case of osteosarcoma in a patient with Albright's syndrome following a marked elevation of growth hormone secondary to a pituitary adenoma.[5] The stimulatory effects of growth hormone have been well documented at the cellular level,[6] and it has also been demonstrated that the proliferation of osteosarcoma cell lines can be inhibited by antagonists of growth hormone-releasing hormone (GHRH).[7,8]

One important difference between our patient and the reports in the literature is the length of time between the start of growth hormone therapy and the development of the osteosarcoma. The period in the two previous reported cases was 18 months and 8 years,[1] respectively, and in Darendeliler *et al.*'s[4] study the gap was just over 4 years. In comparison, our case developed an osteosarcoma after only 6 weeks. This short period makes it unlikely that growth hormone initiated the malignant development of the osteosarcoma.

From a histological perspective, it has been suggested that non-common subtypes of osteosarcoma may be predictive of patients with rare cancer syndromes.[9] However, our patient had a common subtype. Unfortunately the fine histological detail of the previous five cases is not reported.

In conclusion, we present a sixth case of osteosarcoma in transfusion-dependent Diamond–Blackfan anaemia, who is currently alive with pulmonary metastases. This adds further evidence to the association between theses two relatively rare conditions.

### References

1. Lipton JM, Federman N, Khabbaze Y, Schwartz CL, Hilliard, LM, Clark JI, Vlachos A. Osteogenic sarcoma associated with Diamond–Blackfan anemia: a report from the Diamond–Blackfan registry. *J Pediatr Hematol Oncol* 2001; 23: 39–44.
2. Aquino VM, Buchanan GR. Ostoegenic sarcoma in a child with transfusion-dependent Diamond-Blackfan anemia. *J Pediatr Hematol Oncol* 1996; 18: 230–2.
3. Willig T-N, Niemeyer CM, Leblanc T, *et al.* Identification of new prognosis factors from the clinical and epidemiological analysis of a registry of 229 Diamond–Blackfan anemia patients. *Pediatr Res* 1999; 46: 553–61.
4. Darendeliler F, Bundak R, Eryilmaz SK, Gunoz H, Bas F, Saka N. Follow-up height after discontinuation of growth hormone treatment in children with intra-uterine growth retardation. *J Pediatr Endocrinol Metab* 2002; 15: 795–800.
5. Hall MB, Sclar AG, Gardner DF. Albright's syndrome with reactivation of fibrous dysplasia secondary to pituitary adenome and further complicated by osteogenic sarcoma. Report of a case. *Oral Surg Oral Med Oral Pathol* 1984; 57: 616–9.
6. Scheven BA, Hamilton NJ, Fakkeldij TM, Duursma SA. Effects of recombinant human insulin-like growth factor I and II (IGF-I/-II) and growth hormone (GH) on the growth of normal adult human osteoblast-like cells and human osteogenic sarcoma cells. *Growth Regul* 1991; 1: 160–7.
7. Pinski J, Schally AV, Groot K, Halmos G, Szepeshazi K, Zarandi M, Armatis P. Inhibition of growth of human osteosarcomas by antagonist of growth hormone-releasing hormone. *J Natl Cancer Inst* 1995; 87: 1787–94.
8. Brackowski R, Schally AV, Plonowski V, Varga JL, Groot K, Krup M, Armatis P. Inhibition of proliferation in human MNNG/HOS osteosarcoma and SK-ES-1 Ewing sarcoma cell line in vitro and in vivo by antagonists of growth hormone-releasing hormone: effects on insulin-like growth factor II. *Cancer* 2002; 95: 1735–45.
9. Hauben EI, Arends JJ, Vandenbrouke JP, van Asperen C, van Marck E, Hogendoorn, PCW. Osteosarcoma subtypes in osteosarcoma patients with multiple primary malignancies. Can rare subtypes be indicative for cancer syndromes related with osteosarcoma? *Sarcoma* 2002; 6 (Suppl): 13–18 (EMSOS abstract B22).
